# Anti-Metalloprotease P-I Single-Domain Antibodies: Tools for Next-Generation Snakebite Antivenoms

**DOI:** 10.1155/2022/2748962

**Published:** 2022-07-19

**Authors:** Marcela C. S. Silva, Soraya S. Pereira, Marilia P. Gouveia, Marcos B. Luiz, Rosa M. O. Sousa, Anderson M. Kayano, Aleff F. Francisco, Nidiane D. R. Prado, Leandro S. M. Dill, Marcos R. M. Fontes, Fernando B. Zanchi, Rodrigo G. Stabeli, Andreimar M. Soares, Juliana P. Zuliani, Carla F. C. Fernandes

**Affiliations:** ^1^Fundação Oswaldo Cruz, Fiocruz Rondônia, Porto Velho, 76812-245 Rondônia, Brazil; ^2^Centro de Pesquisa em Medicina Tropical, Porto Velho, 76812-329 Rondônia, Brazil; ^3^Departamento de Biofísica e Farmacologia, Instituto de Biociências, UNESP, Botucatu, 18618-689 São Paulo, Brazil; ^4^Plataforma Bi-Institucional Fiocruz-USP, Ribeirão Preto, 14040-030 São Paulo, Brazil; ^5^Instituto Nacional de Ciência e Tecnologia de Epidemiologia da Amazônia Ocidental, INCT-EpiAmO, Brazil; ^6^Universidade Federal de Rondônia, UNIR, Porto Velho, 76801-974 Rondônia, Brazil; ^7^Fundação Oswaldo Cruz, Fiocruz Ceará, Eusébio, 61760-000 Ceará, Brazil

## Abstract

In order to address the global antivenom crisis, novel antivenoms need to present high therapeutic efficacy, broad neutralization ability against systemic and local damage, sufficient safety, and cost-effectiveness. Due to biological characteristics of camelid single-domain antibodies (VHH) such as high affinity, their ability to penetrate dense tissues, and facility for genetic manipulation, their application in antivenoms has expanded considerably. VHHs that are active against the metalloprotease BjussuMP-II from the snake *Bothrops jararacussu* were selected. After isolation of BjussuMP-II, a camelid was immunized with the purified toxin in order to construct the recombinant phage library. Following a round of biopanning, 52% of the selected clones were able to recognize BjussuMP-II in an ELISA assay. After sequencing, seven sequence profiles were identified. One selected clone (VHH61) showed cross-reactivity to *B. brazili* venom, but did not recognize the *Crotalus* and *Lachesis* genera, indicating specificity for the *Bothrops* genus. Through *in vitro* tests, the capacity to neutralize the toxicity triggered by BjussuMP-II was observed. Circular dichroism spectroscopy indicated a robust secondary structure for VHH61, and the calculated melting temperature (*T*_M_) for the clone was 56.4°C. *In silico* analysis, through molecular docking of anti-BjussuMP-II VHHs with metalloprotease, revealed their potential interaction with amino acids present in regions critical for the toxin's conformation and stability. The findings suggest that anti-BjussuMP-II VHHs may be beneficial in the development of next-generation antivenoms.

## 1. Introduction

Snakebites predominantly occur in tropical and subtropical countries located in Africa, Asia, Oceania, and Latin America and affect more than 5 million individuals/year. There are about 2.5 million reported envenoming cases, resulting in 400,000 individuals with permanent sequelae and more than 100,000 deaths annually [1, 2]. The affected individuals are mainly working-age male agricultural workers (15 to 49 years old) [3–5]. In Brazil, approximately 30,000 snakebites were reported in 2018, of which 73.5% were attributed to snakes of the genus *Bothrops*, followed by the genera *Crotalus* (7.5%), *Lachesis* (3.0%), and *Micrurus* (0.7%) [6].

Snake venoms are a complex and variable mixture of more than 100 toxic proteins along with other proteins that, while not toxic, still produce a diverse range of effects [7]. The toxic components of the venom of snakes belonging to the *Bothrops* genus induce significant changes in the site of inoculation through several mechanisms. These local changes are triggered independently or through the association of toxins that act synergistically [8, 9], mainly zinc-dependent snake venom metalloproteinases (SVMPs) and phospholipases A_2_ (PLA_2_s) [10]. Toxicity was observed when myoblasts (C2C12) were treated with both an SVMP and a PLA_2_ from *Bothrops alternatus* [11]. The complexity of the interactions that result in the synergistic effect between SVMPs and PLA_2_s was recently reviewed [12].

SVMPs are responsible for hemorrhagic activity and are associated with PLA_2_-mediated myotoxicity, which evolves rapidly and can cause extensive local tissue damage since it triggers lysis of the plasma membrane and hypercontraction, leading to damage to skeletal muscle cells and, consequently, loss of the affected limb [13]. SVMPs have a molecular mass between 20 and 100 kDa and are dependent on metal ions, mainly zinc (Zn^2+^), for their catalytic activity [14]. These proteins are classified into three groups (P-I through P-III) based on the presence of accessory domains to the proteolytic domain (HEXXHXXGXXH) [15]. SVMPs isolated from Latin American snake venoms cause dermonecrosis, complement activation, fibrinolysis, fibrinogenolysis, and TNF release, all of which are associated with the pathophysiology of envenomation. [13].

The SVMP PI class (20 to 30 kDa) is composed solely of the proteolytic domain and can induce vascular permeability [16], edema [17], and influx of leukocytes *in vitro* [18] and *in vivo*, resulting in an increase in the number of these cells in the blood. They are associated with specific expression of leukocyte adhesion molecules and the release of inflammatory chemotactic cytokines, demonstrating that only the proteolytic domain of metalloproteases may trigger certain inflammatory events [19].

BjussuMP-II, a PI class metalloproteinase, was isolated from *B. jararacussu* venom. With a molecular mass of 24 kDa, this metalloprotease demonstrated the ability to hydrolyze the *α* chain and, to a lesser extent, the *β* chain of fibrinogen, thus being classified as an *α*/*β*-fibrinogenase. Its activity was enhanced when incubated with Zn^2+^ and Ca^2+^ ions and inhibited when incubated with heparin and EDTA. BjussuMP-II exhibits inhibitory activity on platelet aggregation induced by ADP (adenosine diphosphate) and collagen. Despite being considered nonhemorrhagic when compared to venom, its histopathological analysis showed the presence of focal leukocyte infiltrates, slight destruction of muscle fibers and hemorrhaging in lung tissue [20].

The recommended treatment for snake envenoming is serum therapy, consisting of polyclonal antibodies or antibody fragments (F(ab) or F(ab′)2) derived from the plasma of hyperimmunized animals [2]. Although this therapy is effective against systemic damage, it has limitations in its treatment of local tissue damage, often resulting in permanent motor disability [19]. In addition, the induction of adverse reactions by some serum therapeutic agents and the need for more efficient, homogeneous, and safe preparations have instigated the search for alternative processes and products for the treatment of snake envenoming [21].

A variety of studies have demonstrated the ability of IgG and camelid single-domain antibodies (VHH or nanobodies) from venom-immunized camels and llamas to neutralize animal toxins with the hopes of developing new antivenoms [22–29]. The single-domain antibodies anti-*α*-cobratoxin (*α*Cbtx) and anti-PLA_2_ from *Naja kaouthia* showed recognition and neutralization capacity *in vitro* [24, 25] and *in vivo* [26]. Furthermore, selected nanobodies were able to neutralize toxins from *Bothrops* and *Crotalus* snake venoms (BthTX-I and BthTX-II from *Bothrops jararacussu*, crotoxin from *Crotalus durissus terrificus*, and metalloprotease and PLA_2_ from *Bothrops atrox*) *in vitro* and *in vivo* [27–29].

Faced with the need to develop more efficient antiophidic therapeutic formulations with ample capacity to neutralize toxins, with less immunogenicity and at a lower cost, single-domain antibodies stand out for their potential as innovative therapeutic agents against snake envenoming [30–32]. They are also useful in biomedical research, such as in bioprospecting for toxins, or in laboratorial diagnosis of snake envenoming. Accordingly, this study is aimed at selecting single-domain antibodies able to recognize the metalloprotease BjussuMP-II isolated from *B. jararacussu*.

## 2. Materials and Methods

### 2.1. Ethical Considerations

All experimental procedures involving animals were performed in accordance with the recommendations of the National Council for the Control of Animal Experiments (CONCEA) and were approved by the Ethics Committee in the Use of Animals at Fiocruz Rondônia, under the 2012/11 protocol. The license related to access to Brazilian genetic resources for scientific purposes used in the proposed activities is registered with the National Management System of Genetic Heritage and Associated Traditional Knowledge (SISGEN) under registration number A444D6C. The authorization for handling Genetically Modified Organisms (GMOs) and their derivatives are also registered according to CQB 319/15-Fiocruz Rondônia.

### 2.2. Isolation and Characterization of BjussuMP-II

Approximately 100 mg of desiccated *B. jararacussu* venom was solubilized in 1 mL of 50 mM ammonium bicarbonate (NH_4_HCO_3_) (AMBIC), pH 8.0 [20]. A CM-Sepharose FF® column (GE Healthcare Lifesciences, USA) (1.0 × 40.0 cm) was used to fractionate the venom (100 mg), previously equilibrated with the same 500 mM AMBIC buffer, pH 8.0, in 5 column volumes, at a flow of 1 mL/min, in an Akta Purifier® chromatography system (GE Healthcare Life Sciences, USA). The eluted fractions were monitored at 280 nm and were collected manually. Sample purity was assessed using electrophoretic analysis on a 12.5% polyacrylamide gel under denaturing conditions (SDS-PAGE 12.5%), as described by [33], and stained with Coomassie Blue (Contemporary Chemical Dynamics, Brazil) in order to visualize the protein bands.

The mass spectrum was obtained in an AXIMA/TOF2 Mass Spectrometer (Shimadzu Biotech). A saturated solution of sinapinic acid (Sigma, USA) was used as the ionizing matrix, homogenized with the sample at a ratio of 1 : 1 (matrix/protein), and after cocrystallization in the device plate, this sample was introduced into the chamber vacuum and analyzed in linear mode.

To verify fibrinogenolytic activity of purified BjussuMP-II, bovine fibrinogen samples (200 *μ*g) were incubated with 10 *μ*g of *B. jararacussu* venom and, separately, with 10 *μ*g of BjussuMP-II, at 37°C for a period of 30 min, 1, 3, and 6 h. The reaction was stopped by adding 0.5 mM Tris-HCl buffer, pH 8.0 containing 20% glycerol (*v*/*v*), 4% SDS (*m*/*v*), 0.05% bromophenol blue (*m*/*v*), and 0.3% DL-dithiothreitol (*w*/*v*), at a ratio of 1 : 1 (*v* : *v*) [34]. Then, the fibrinogen digests were analyzed on 12.5% SDS-PAGE (*m*/*v*), stained with a solution containing 0.08% Coomassie Brilliant Blue G-250® (*m*/*v*), 8% aluminum sulfate (*m*/*v*), 1.6% o-phosphoric acid (*m*/*v*), and 20% methanol (*v*/*v*) for 15 min. Excess dye was removed by immersing the gel in a bleaching solution containing 4.0% ethanol and 7% (*v*/*v*) acetic acid in water. The gel image was obtained using Image scanner® (GE Healthcare Life Sciences) equipment.

### 2.3. Immunization of a Camelid and Evaluation of Its Immune Response

A young adult male *Lama glama*, with food and water *ad libitum*, was immunized at weekly intervals five times with 200 *μ*g of BjussuMP-II, subcutaneously, in addition to complete (first dose) and incomplete Freund's adjuvant (subsequent doses). In order to monitor the immune response, ELISA plates were adsorbed with 100 *μ*L of BjussuMP-II (10 *μ*g/mL) diluted in PBS (phosphate-buffered saline). After incubation for 12 h at 4°C, the plates were washed 3 times with 0.05% PBS Tween 20 (PBST) (ELx50®, BioTek) and the nonspecific sites were blocked for 5 h with 250 *μ*L of blocking solution (5% skim milk in PBS). After washing, the serum of the immunized animal was diluted to 1 : 10^2^, 1 : 500, 1 : 10^3^, 1 : 5.000, 1 : 10^4^, 1 : 50.000, and 1 : 10^5^ in blocking solution (BS) and 100 *μ*L was added to each well, being incubated for 12 h at 4°C. After washing, rabbit anti-llama IgG_2_/IgG_3_ antibody [35] diluted to 1 : 12,000 in BS was added to each well and incubated at 4°C for 12 h. After washing the plates again, 100 *μ*L per well of anti-rabbit IgG produced in mice conjugated with peroxidase (Sigma-Aldrich, USA) was added at a dilution of 1 : 40,000 in BS and incubated for 4 h. The reaction was developed with 100 *μ*L/well of tetramethylbenzidine (TMB, Millipore, USA), after washing with PBST. After 30 min, the reaction was stopped with the addition of 100 *μ*L of 0.32 M sulfuric acid and absorbances were measured in a spectrophotometer (BioTek-Synergy HT, USA) at a wavelength of 450 nm. Samples were tested in triplicate. The negative control corresponded to uncorrelated serum (1 : 10^2^ camelid serum immunized with recombinant hantavirus protein—rN*Δ*85).

### 2.4. Construction of the VHH Library and Selection of Anti-BjussuMP-II VHHs

Three days after the final booster, 50 mL of blood was collected from the animal, and lymphocyte isolation was performed with Ficoll-Paque PLUS® (Amershan Biosciences, UK). Total RNA extraction from lymphocytes was performed with the aid of Trizol Reagent® (Invitrogen, USA) and cDNA synthesis was performed with the SuperScript III first strand synthesis system for RT-PCR (Invitrogen, USA). The constructed cDNA was submitted to polymerase chain reaction (PCR) using gene-specific oligonucleotides, as described by [36]. The resulting amplification products from the second PCR were recombined in a pHEN-1 phagemid vector. Subsequently, the recombined phagemids were transformed into an electrocompetent TG1 strain of *E. coli* bacteria. The genetic repertoire of VHHs, as well as an immune library, and recombinant phages expressing VHHs fused to protein III of helper phage M13K07 were obtained according to [27].

Selection of anti-BjussuMP-II VHHs was performed using biopanning. For this, immunotubes (Nunc-MaxiSorp/Thermo Scientific, USA) were adsorbed with 1 mg of BjussuMP-II diluted in PBS. Excess BjussuMP-II was washed three times with PBST at pH 7.4 and blocked with BS for 4 h. The phage library (1.8 × 10^11^ recombinant phage particles) expressing the VHH repertoire, previously incubated in BS, was added to the immunotube and incubated under shaking conditions for 30 min, followed by 1 h and 30 min with no shaking at room temperature. Then, elution of phages was performed with 100 mM of HCl, and neutralized with 1 M Tris-HCl, pH 7.5. Selected phages were transferred to TG1 *E. coli* (OD600 = 0.5) and incubated at 37°C. After centrifuging and discarding the supernatant, the pellet was resuspended in 2YT culture medium, seeded in 2YT/amp/glu plates, and incubated for 15 h at 30°C. The presence of VHHs in selected clones was verified by colony PCR and positive clones were selected to verify antigen-binding specificity in an ELISA assay. The negative control corresponded to uncorrelated serum (1 : 1000 *v*/*v* alpaca serum immunized with recombinant hantavirus protein—rN*Δ*85). The clones that showed the best reactivity in ELISA were subjected to plasmid DNA extraction using a QIAprep Spin Miniprep kit (Quiagen, USA), according to the manufacturer's instructions. Subsequently, the material was sequenced on the DNA Sequencing Platform from Fiocruz-MG (DNA Sequencer—ABI 3730 from Life Technologies).

### 2.5. Expression and Purification of Anti-BjussuMP-II VHHs

A representative of each of the seven nanobody profiles was subcloned into a pET-22b(+) vector, aiming at soluble expression. For this, polymerase chain reaction (PCR) was performed using plasmid DNAs as templates and gene-specific primers designed with restriction enzyme sites (*Nde*I and *Xho*I): VHNDEF′: 5′GGAATTCCATATGGCCGA(G/C)GT(G/C)3′ and VHXHOR′: 5′CCGCCTCGAGTGAGGAGACGG3′. For the PCR assay, 100 ng of plasmid DNA was added to a mix containing 5 *μ*L 10× buffer, 1 *μ*L each primer (0.2 *μ*M), 1 *μ*L dNTP mix (2.5 mM each), 0.25 *μ*L Hotmaster Taq Polymerase enzyme (1.25 U) (5 Prime, Germany), and water to a final volume of 50 *μ*L. Anti-hanta VHH36 plasmid DNA was used as a positive control [35], and negative control was performed without plasmid DNA. The PCR amplification consisted of 30 cycles (denaturation for 30 seconds at 94°C, annealing for 30 seconds at 56°C, extension for 30 seconds at 72°C). The final extension step was carried out at 72°C for 10 minutes. Subsequently, PCR products were digested with the endonucleases *Nde*I and *Xho*I (New England BioLabs, Germany), following manufacturer's instructions, and recombination using pET-22b(+) vector was performed, as described by Luiz et al. [28]. Then, the clones were transformed and cultivated in BL21(DE3) *E. coli*, in an Erlenmeyer flask containing 10 mL of Luria-Bertani (LB) medium containing 50 *μ*g/mL of ampicillin (LB-amp) and incubated under shaking conditions (200 rpm) at 30°C for 16 h. About 1% of the preinoculum volume was transferred to 500 mL of LB-amp medium (50 *μ*g/mL ampicillin) and maintained under agitation (200 rpm) at 30°C, until reaching an optical density of 1.0 in a waveform length of 600 nm (OD600 nm = 1.0). The inoculum was induced with isopropyl-d-1-thiogalactopyranoside (IPTG) at a final concentration of 0.3 mM for 20 h under agitation (200 rpm) at 25°C. Subsequently, the culture was centrifuged for 15 min at 4,500 rpm at room temperature and the bacterial pellet was resuspended in 25 mL of lysis buffer (10 mM Tris base, 1 M NaCl, Triton X-100 (0.05%, pH 8.0)). Cellular contents were lysed on ice through 2 cycles of ultrasonication (Vibracell ® VC100, Sonic and Materials Inc.) with an interval of 30 s, adapted from Dantas et al. [37]. Bacterial lysates were centrifuged for 15 min at 10,500 rpm in order to separate soluble contents from insoluble cell debris.

Affinity purification was performed on a chromatographic column of cobalt coupled to agarose matrix (GE Healthcare, USA), using a peristaltic pump (Pump P-1, GE Healthcare, USA) with a flow of 1 mL/min. Affinity chromatography was performed as described in the Talon superflow manual under native conditions, using phosphate buffer (anti-BjussuMP-II VHH 47) and using the protocol adapted from Ding et al. [38] for purification (anti-BjussuMP-II VHHs 61 and 64). The eluate was subjected to diafiltration on 3 kDa Amicon columns (Millipore, USA) at 4,000 × *g* for 20 min at 16°C for four cycles using the binding buffer for sample buffer exchange and imidazole removal. The amount of recovered protein was quantified using the Smith method [39] with a BCA Protein Assay kit (Pierce™, Thermo Scientific), and readings were performed in a spectrophotometer at a wavelength of 562 nm (BioTek-Synergy-HT, USA).

### 2.6. Evaluation of Anti-BjussuMP-II VHHs' Reactivity in an Immunoenzymatic Assay (ELISA)

An ELISA-type immunoenzymatic assay was performed in order to evaluate the immunoreactivity of the VHHs against BjussuMP-II. For this purpose, Nunc-MaxiSorp plates (eBioscience, USA) adsorbed with 1 *μ*g of BjussuMP-II diluted in PBS buffer (*v*/*v*) and incubated for 24 h at 4°C were used. Subsequently, the wells were blocked with BS and incubated at 4°C overnight. One *μ*g of anti-BjussuMP-II VHHs diluted in BS was applied and incubated at 4°C for 24 h. Then, the plate was washed three times, and 100 *μ*L of rabbit anti-llama IgG_2_/IgG_3_ antibody [35] diluted in BS at a ratio of 1 : 1 × 10^3^ (*v*/*v*) was incubated at 4°C for 24 h.

The wells were washed again, and then, 100 *μ*L of peroxidase-conjugated anti-rabbit IgG secondary antibody (Sigma-Aldrich, USA) previously diluted to 1 : 5 × 10^3^ (*v*/*v*) in BS was applied and the plate was incubated at 4°C for 4 h. The reaction was developed with 100 *μ*L of tetramethylbenzidine (TMB, Millipore, USA), and absorbances were measured at a wavelength of 450 nm. One *μ*g of anti-Hanta36 VHH specific for hantavirus [35] was used as an uncorrelated negative control-UN, and the positive control consisted of the minimum dilution of the reagent serum of the animal immunized with the toxin (1 : 10^4^ *v*/*v*). The cut-off line (cut-off value) was established as twice the mean of the negative control's absorbances plus twice the sample's standard deviation (2 × negative mean + 2 × SDSD).

### 2.7. Evaluation of Anti-BjussuMP-II VHH Cross-Reactivity for Snake Venoms and Toxins by ELISA

For this immunoassay, 1 *μ*g of different toxins and venoms from *Bothrops*, *Crotalus*, and *Lachesis* genera diluted in 1× PBS buffer was adsorbed in the wells of the plate. After blocking and washing, 1 *μ*g of anti-BjussuMP-II VHH 61 was added to BS and incubated at 4°C for 24 h. Then, after washing the plate, 100 *μ*L of anti-His (GE Healthcare Life Sciences, Little Chalfont, BKM) produced in mice was added and diluted to a ratio of 1 : 1000 (*v*/*v*) in blocking solution and the plate was incubated at 4°C for 24 h.

Then, washing was performed and 100 *μ*L of peroxidase-conjugated mouse anti-IgG produced in goats (Southern Biotech, Birmingham, AL) was added at a dilution of 1 : 5,000 (*v*/*v*) in blocking solution; then, the plates were incubated at 4°C for 4 h. After washing the plate again, 100 *μ*L of tetramethylbenzidine (TMB, Millipore, USA) was added for development, with an incubation period of 30 min, followed by the addition of 100 *μ*L of 0.32 M sulfuric acid which interrupted the reaction. The negative control employed was the VHH anti-Hanta36, and the positive control reaction consisted of recognizing anti-BjussuMP-II VHH 61 with wells adsorbed with previously tested BjussuMP-II. Samples were tested in duplicate. The data were tabulated in the program GraphPad Prism 5 2007®, and the “cut-off” was established as (2 × StandDev + 2 × Mean *C*−).

### 2.8. Inhibition of BjussuMP-II Proteolytic Activity

The proteolytic activity of BjussuMP-II on casein was evaluated according to the method described by Cupp-Enyard [40] with modifications. For the assay, the protease (10 *μ*g) was incubated for 10 min at 37°C with a 1% casein suspension in 0.1 M Tris-HCl pH 9.0. After the incubation period, 500 *μ*L of 110 mM TCA was added, followed by incubation at 37°C for 30 min and centrifugation at 16,000 × *g* for 30 min. The supernatant was filtered with 0.22 *μ*m pore membranes, and a 200 *μ*L aliquot was incubated with 500 *μ*L of 500 mM sodium carbonate and 100 *μ*L of 0.5 M Folin reagent (Dynamics) at 37°C for 30 min. Finally, 200 *μ*L of the samples was added to a 96-well microtiter plate and the optical density was determined at a wavelength of 660 nm using a spectrophotometer (Eon, BioTek). 1% casein solution was used as a blank.

For the proteolytic activity inhibition assays, BjussuMP-II (10 *μ*g) was preincubated at 37°C for 1 h with anti-BjussuMP-II VHHs in different proportions (1 : 0.25; 1 : 0.5; 1 : 1; 1 : 2; 1 : 3; 1 : 4; 1 : 5; 1 : 10; 1 : 20; and 1 : 40 molar ratios). BjussuMP-II was used as a positive control of enzymatic activity, and 1% casein solution was used as a negative control. After this preincubation period, proteolytic activity was determined as described above. All experimental conditions were performed in triplicate, and the results were expressed as absorbance measurements (660 nm) of the proteolytic activity as compared to the positive control.

### 2.9. In Vitro Inhibition of BjussuMP-II Cytotoxicity

Cultivation of murine endothelial cells (EC) of the tEnd lineage (thymic endothelium) was performed according to Franco et al. [41]. Beginning in 75 cm^2^ polystyrene bottles (COSTAR), the cells were maintained in 20 mL of DMEM culture medium, supplemented with 10% fetal bovine serum (FBS) and antibiotic (gentamicin 80 mg/mL) in a humidified atmosphere of 5% CO_2_ at 37°C, until a confluent EC monolayer was obtained, with renewal of the culture medium every 48 h. From the subculture, the viable cells were seeded in Costar® 96-well microplates (Sigma, USA), in a proportion of 2 × 10^5^ CE/well, and incubated in a humid atmosphere at 5% CO_2_ and 37°C, for 24 h. Dilutions of BjussuMP-II and anti-BjussuMP-II VHH61 were carried out in serial concentrations from 20 *μ*g to 0.31 *μ*g of BjussuMP-II and from 100 *μ*g to 1.55 *μ*g of anti-BjussuMP-II VHH61 (1 : 5 mass/mass ratio). EC were incubated with BjussuMP-II and/or VHH, diluted in DMEM. Untreated cells were used as the negative control, and cells treated with 0.1% Triton X-100 solution were used as the positive control. Assays were performed as described by Franco et al. [41], with modifications, at 1, 24, and 48 h.

The enzymatic activity of LDH present in the medium was determined as a parameter of cell viability. After the treatment times, the supernatant was removed and 10 *μ*L was added to a Corning® UV microplate (Sigma, USA), accompanied by the addition of 250 *μ*L of the pyruvate substrate, in phosphate buffer containing 200 mM NaCl, 0.2 mM NADH, and 1.6 mM pyruvate/well followed by two kinetic readings in a microplate spectrophotometer (Biochrom Asys, Expert Plus, Holliston, USA) at a wavelength of 340 nm, with 1 min intervals at 37°C, as per the manufacturer's specifications (Labtest, Brazil). The results were expressed as the decrease in O.D., resulting from the oxidation of NADH by pyruvate, in relation to the negative control.

### 2.10. Investigation of Anti-BjussuMP-II VHH61's Secondary Structure by Circular Dichroism Spectroscopy

Circular dichroism (CD) spectroscopy experiments were performed at 20°C in a J-815 spectropolarimeter (Jasco Inc., Tokyo, Japan) to evaluate anti-BjussuMP-II VHH61. Anti-BjussuMP-II VHH61 was solubilized in 10 mM phosphate buffer pH 7, filtered (using PVDF filters with 0.22 *μ*m diameter pores) and quantified in a NanoDrop 2000C spectrophotometer (Thermo Fisher Scientific, Waltham, USA). The CD spectrum corresponds to an average of 40 accumulations obtained with a resolution of 0.5 nm and a bandwidth of 2 nm, within a range of 191-260 nm at 100 nm/min scanning speed. All the CD spectra collections were performed in the presence of a constant flow of N_2_ gas, in order to avoid O_2_ interference in the measurements. CD spectra data were corrected for the solvent and normalized to mean residue ellipticity (MRE and/or (*θ*)) from optical path length, molar concentration (6.67 *μ*M), and primary structure length of anti-BjussuMP-II VHH61, using Spectra Analysis software (Jasco Inc., Tokyo Japan). Deconvolution of the normalized CD spectra was performed with the BeStSel [42] algorithm; the *K*-nearest neighbor search using the chain length was performed for fold prediction. A moderate smoothing using the Savitzky-Golay filter was applied to all experimental data points which were further plotted using Origin version 8.0 (OriginLab Corporation, Northampton, MA, USA).

Thermal denaturation analysis was performed by monitoring the circular ellipticity changes at a fixed wavelength of 202 nm, while the sample was heated from 20 to 90°C. Data points were acquired by a ramp rate of 1°C/min and an equilibration time of 60 s after each temperature adjustment using an optical path length of 2.0 mm. The denaturation curve was normalized and fitted employing the standard two-state model *N*⇌*D* (*N* for native and *D* for denatured) according to the equation *y*(*ξ*) = *f*_*N*_(*a*_*N*_ + *b*_*N*_*ξ*) + *f*_*D*_(*a*_*D*_ + *b*_*D*_*ξ*), where *f*_*N*_ and *f*_*D*_ are the fractional populations of the two states, *ξ* is the temperature, and (*a*_*i*_ + *b*_*i*_*ξ*) is the signal for state *i*, and the melting temperature (*T*_M_) was calculated and plotted with CDpal implementation [43].

### 2.11. Modeling and Molecular Docking of Anti-BjussuMP-II VHHs

Modeling of the selected anti-BjussuMP-II VHHs (34, 47, 53, 61, 64, 78, and 79) and BjussuMP-II were performed using Protein BLAST software [44] (http://blast.ncbi.nlm.nih.gov), ClustalW [45] (https://ebi.ac.uk/) for sequence search and alignment, and the Protein Data Bank (PDB) (http://www.pdb.org) for template retrieval. The software Modeller v.9.10 [46] (https://salilab.org/modeller/) was used for structural construction and the best model selected was the one with the lowest score in the DOPE energy criterion. Model validation was performed using the software Procheck [47] and Verify3D [48, 49] (https://saves.mbi.ucla.edu/). The overlap between the model and the mode to calculate the root mean square deviation (RMSD) was also performed. Interactive visualization and comparative analysis of molecular structures were performed using Swiss-PDB viewer v4.1 [50] (https://spdbv.vital-it.ch/) and USCF Chimera [51] software. The ClusPro2.0 server (http://ClusPro.bu.edu/) was used for the evaluation of interaction and possible intermolecular orientation. In this software, a docking assay was performed, selecting the VHH as the receptor and the toxin as the ligand in the “antibody mode” of the platform.

## 3. Results

### 3.1. Purification and Characterization of BjussuMP-II

**Figure 1:**
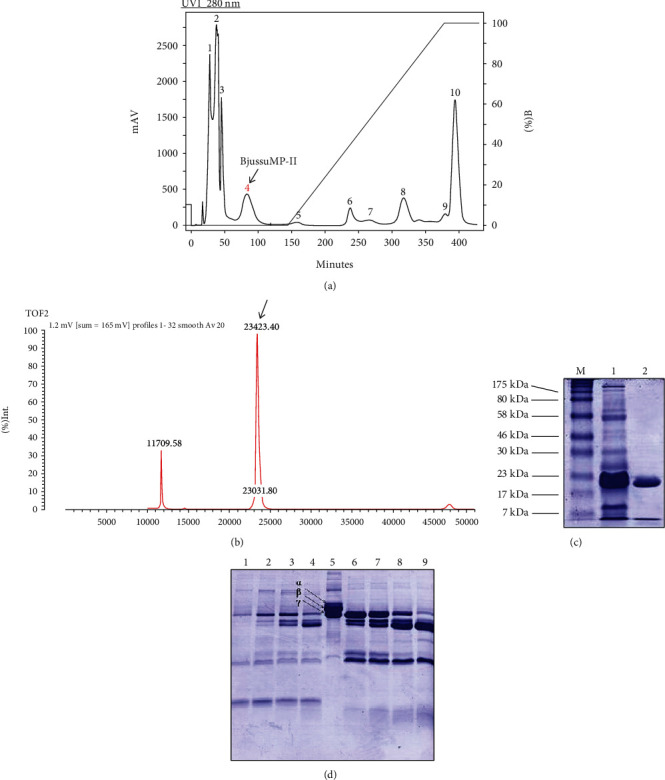
Purification and characterization of BjussuMP-II. (a) Chromatographic profile of *B. jararacussu* venom using cation exchange chromatography. Fraction 4 was selected for presenting a relative molecular mass of 24 kDa in electrophoretic analysis on a 12.5% polyacrylamide gel. (b) Polyacrylamide gel electrophoresis (12.5%) of the fraction corresponding to the metalloprotease isolated from *B. jararacussu* venom (1) and of the purified metalloprotease BjussuMP-II, with an approximate molecular mass of 24 kDa (2). (c) Mass spectrum of BjussuMP-II in AXIMA TOF2. The spectrum measures the mass of the protein BjussuMP-II, at 23,423.40 Da. (d) Electrophoretic analysis of the fibrinogenolytic activity of *B. jararacussu* venom and BjussuMP-II at different incubation times, for 30 min (6), 1 h (7), 3 h (8), and 6 h (9). The negative control corresponds to fibrinogen (5).

Chromatography of *B. jararacussu* venom on a CM Sepharose FF® cation exchange column with AMBIC pH 8.0 buffer resulted in 10 main fractions (Figure 1(a)), of which fraction 4 was obtained with a retention time of approximately 80 min and was selected based on its relative molecular mass of 24 kDa as shown in SDS-PAGE (Figure 1(b)). The selected fraction 4 yielded about 5 mg, 5% from 100 mg of *B. jararacussu* venom used for chromatography. Mass spectrometry of fraction 4 revealed the presence of a single constituent measuring 23,423.40 Da and a corresponding double charge of 11,790.58 Da, corroborating the relative mass visualized in the one-dimensional electrophoresis gel (Figure 1(c)).

Furthermore, both *B. jararacussu* venom and BjussuMP-II were able to completely hydrolyze fibrinogen *α* and *β* chains after 30 min of incubation and showed activity against fibrinogen *γ* chain, which can be best observed after 6 h of incubation (Figure 1(d)).

### 3.2. Follow-Up of the Camelid Immune Response and Selection of Anti-BjussuMP-II VHHs

**Figure 2:**
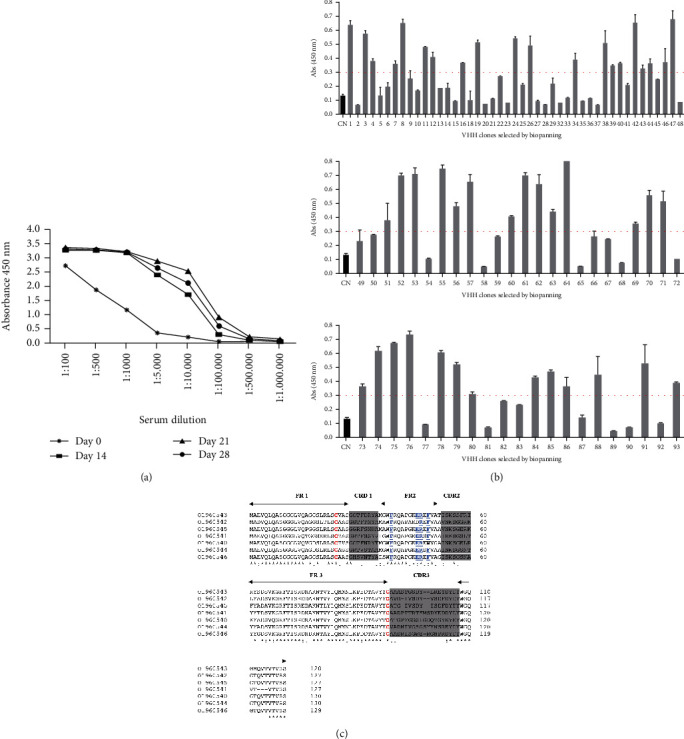
Identification of anti-BjussuMP-II VHHs. (a) Monitoring of the camelid immune response after administration of BjussuMP-II. Results obtained in an ELISA assay, demonstrating the absorbances obtained in different dilutions of the serum collected on the following days: 0 (∗), 14 (■), 21 (▲), and 28 (•). (b) Analysis of VHHs against the toxin BjussuMP-II after biopanning. The cut-off line corresponds to the average of the cut-off values calculated for each of the 47 clones. (c) Multiple alignments among clones selected against BjussuMP-II, demonstrating CDRs and FRs. Letters highlighted in red represent the cysteines present in all clones. The gray shaded areas represent the CDRs. Amino acids underlined in the FR2 region represent the characteristic amino acids of VHHs. (∗) represents identical amino acids; (:) represents strongly similar amino acids; (.) represents weakly similar amino acids.

The animal's immune response was monitored by ELISA, obtaining a maximum titer of 1 : 500,000 of the responsive serum dilution on the 28^th^ day after the animal's initial immune response (Figure 2(a)). After construction of a recombinant phage library, a single round of biopanning, 90 clones were randomly selected for the ELISA assay, of which 47 clones recognized BjussuMP-II (52%). Ninety clones were submitted to ELISA against the toxin BjussuMP-II. Forty-seven clones recognized the toxin. The negative control corresponds to the absorbance of the wells where the protein was adsorbed together with a dilution (1 : 1000) of the uncorrelated serum. The negative control and clones 01 to 93 are shown in (Figure 2(b)). After sequencing the clones with the best reactivity for BjussuMP-II in the ELISA assay, multiple alignments were performed among the sequences. Seven distinct anti-BjussuMP-II VHH sequence profiles were identified (34, 47, 53, 61, 64, 78, and 79), and the presence of four characteristic amino acid substitutions in the framework region 2 (FR2) of the camelid heavy chain antibody (Y/F37) (E,Q44) (R45) (G/F/L47) was verified, except for clone VHH53, which did not show any modification at residue 44. Clones VHH34 and VHH47 had the most extensive complementarity-determining region 3 (CDR3) with 19 amino acids, followed by clone VHH79 with the presence of 18 amino acids, VHH61 and VHH64 with 17 residues, and clones VHH53 and VHH78 with 16 residues each (Figure 2(c)). Nucleotide sequences of anti-BjussuMP-II VHHs were deposited in the GenBank database under the following accession numbers: VHH34—OL960540, VHH47—OL960541, VHH53—OL960542, VHH61—OL960543, VHH64—OL960544, VHH78—OL960545, and VHH79—OL960546.

### 3.3. Evaluation of VHHs' Reactivity in an Immunoenzymatic Assay (ELISA)

**Figure 3:**
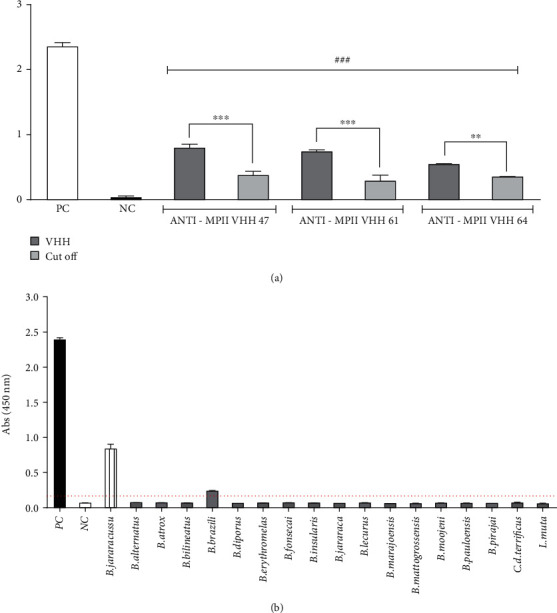
Evaluation of anti-BjussuMP-II VHHs' immunoreactivity by ELISA assay. (a): Absorbances obtained in ELISA performed on plates sensitized with 10 *μ*g/mL of BjussuMP-II and anti-MP-II VHHs in blocking solution (10 *μ*g/mL). PC: positive control with serum from the immunized animal. NC: uncorrelated VHH negative control (anti-Hanta 36 VHH). Columns marked with an asterisk (∗) represent statistical difference in relation to the sample cut-off (^∗∗^*p* < 0.01 and ^∗∗∗^*p* < 0.001). Columns marked with hash (#) represent statistical difference between the nanobodies and the negative control (^###^*p* < 0.001). (b) Absorbances obtained in an ELISA assay performed on a plate sensitized with 1 *μ*g/well of snake venoms of the genera *Bothrops*, *Crotalus*, and *Lachesis*, using a 1 : 1 ratio (*p*/*p*) of anti-BjussuMP-II VHH61. The positive control, shown as a striped bar, stands out above the average cut-off value, represented by a dashed line. The specificity of anti-BjussuMP-II VHH61 for the crude venom of *B. jararacussu* is observed. PC: positive control that corresponds to the absorbance of wells showing the interaction of anti-BjussuMP-II VHH61 with BjussuMP-II from *B. jararacussu*. NC: negative control corresponding to the absorbance of wells containing BjussuMP-II with uncorrelated anti-Hanta36 VHH obtained from *Lama glama*, specific for hantavirus.

Based on expression tests performed with the seven profiles of VHH clones, a low expression profile and yield of VHH34, VHH53, VHH78, and VHH79 were observed, while the following VHHs were selected for the next steps due to their higher yield: VHH47, VHH61, and VHH64. After purification of the VHHs through affinity chromatography on a cobalt column, the reactivity of these clones against BjussuMP-II was evaluated using ELISA. Clones VHH47, VHH61, and VHH64 presented absorbances of 0.9, 0.8, and 0.6, respectively, as measured by spectrophotometry at a wavelength of 450 nm and exhibited immunoreactivity against the toxin BjussuMP-II (Figure 3(a)).

In addition, the evaluation of cross-immunoreactivity in ELISA was performed using the venoms of snakes from *Bothrops*, *Lachesis*, and *Crotalus* genera, after evaluating their reactivity against the isolated toxin BjussuMP-II using ELISA. Anti-BjussuMP-II VHH61 was able to specifically recognize the venom of the snake *B. jararacussu*, and to a lesser extent, the crude venom of *B. brazili*, while it did not recognize the venom of other snakes belonging to other genera, demonstrating its genus specificity (Figure 3(b)).

### 3.4. Inhibition of Proteolytic Activity

**Figure 4:**
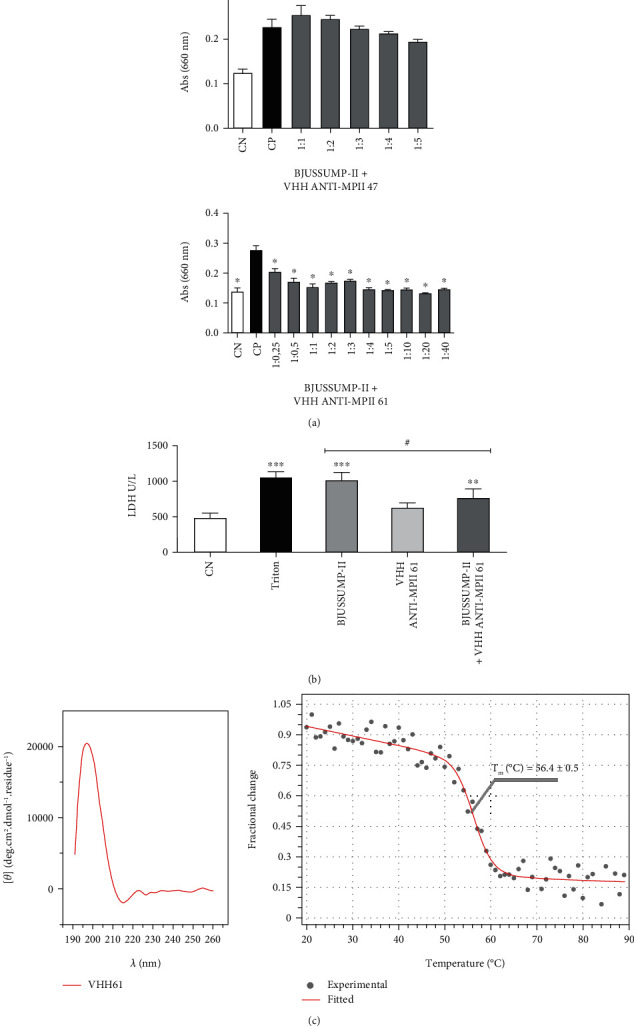
Analysis of inhibition of proteolytic activity and cytotoxicity of BjussuMP-II by anti-BjussuMP-II VHHs, and circular dichroism of anti-BjussuMP-II VHH61. (a) Proteolytic activity of BjussuMP-II on casein after exposure to a preincubated solution containing the toxin and the VHHs (anti-BjussuMP-II VHH47 and anti-BjussuMP-II VHH61) in different molar ratios. NC: negative control consisting of casein and buffer. PC: casein incubated with 10 *μ*g of BjussuMP-II. Columns marked with an asterisk (∗) represent a statistical difference in relation to the positive control (^∗^*p* < 0.001). (b) Analysis of the inhibition of LDH release by tEND cells against exposure to BjussuMP-II by anti-BjussuMP-II VHH61. Cytotoxicity estimated by LDH release after 48 h of exposure to a preincubated solution containing BjussuMP-II and anti-BjussuMP-II VHH61 at a 1 : 5 ratio (*w*/*w*). NC: negative control consisting of untreated cells. Triton: cells treated with Triton X-100 at a concentration of 0.1%. Columns marked with an asterisk (∗) represent a statistical difference in relation to the negative control (^∗∗∗^*p* < 0.001 and ^∗∗^*p* < 0.01). Columns marked with a pound sign (#) represent a statistical difference between BjussuMP-II and BjussuMP-II+anti-BjussuMP-II VHH 61 (^#^*p* < 0.05). (c) Analysis of anti-BjussuMP-II VHH61 circular dichroism spectra collected in the 191-260 nm interval and thermal denaturation curve obtained at a wavelength of 202 nm during heating from 20 to 90°C. The measurements were done in a JASCO J-815 spectropolarimeter.

Spectrophotometry at 660 nm was used to assess the capacity of the chosen VHHs to suppress the proteolytic activity of BjussuMP-II on casein. Simultaneously, the absorbance of preincubated solutions with various concentrations of anti-BjussuMP-II VHH and BjussuMP-II was determined. The results indicated that anti-BjussuMP-II VHH47 had no inhibitory effect on the proteolytic activity of BjussuMP-II in all examined interactions, while anti-BjussuMP-II VHH61 did present inhibition (Figure 4(a)).

### 3.5. In Vitro Inhibition of BjussuMP-II Cytotoxicity

The cytotoxic activity of BjussuMP-II on tEND cells was verified based on the cellular release of LDH in relation to the positive control (cells treated with Triton X-100) and the negative control (cells in DMEM medium) after 48 h (Figure 4(b)). The ability to neutralize the cytotoxic effect of BjussuMP-II on tEND cells by anti-BjussuMPII VHH61 was verified using a preincubated solution at a 1 : 5 weight/weight ratio (toxin/VHH).

After 48 h of incubation, it was possible to determine the apparent cytotoxicity of BjussuMP-II, since there was a release of 1015.68 U/L of LDH by endothelial cells incubated with the toxin. The results demonstrate a significant reduction in the levels of the released LDH (25%, *p* < 0.05) compared to the positive control of treated cells containing only BjussuMP-II. It is also possible to observe that the VHH at the concentration tested (100 *μ*g) did not show cytotoxicity towards the endothelial cells.

### 3.6. Investigation of Anti-BjussuMP-II VHH61's Secondary Structure by Circular Dichroism Spectroscopy

The CD spectra for anti-BjussuMP-II VHH61 revealed an organization of secondary structures with high *β*-sheet content (44.7%: 3.3% left-twisted, 29.2% relaxed, and 12.2% right-twisted), 5.3% *α*-helix, and 11.2% turns. Furthermore, a weighted *K*-nearest neighbor search of anti-BjussuMP-II VHH61's structural domains suggests a sandwich architecture and immunoglobulin-like topology. Overall, anti-BjussuMP-II VHH61's CD spectrum is in complete agreement when compared with the theoretical structural model generated for anti-BjussuMP-II VHH61's sequence (4.5% *α*-helix, 52.2% *β*-strand, and 16.4% turn). The calculated melting temperature (*T*_M_) for anti-BjussuMP-II VHH61 was 56.4°C (Figure 4(c)).

### 3.7. Modeling and Molecular Docking of the Anti-BjussuMP-II VHHs

**Figure 5:**
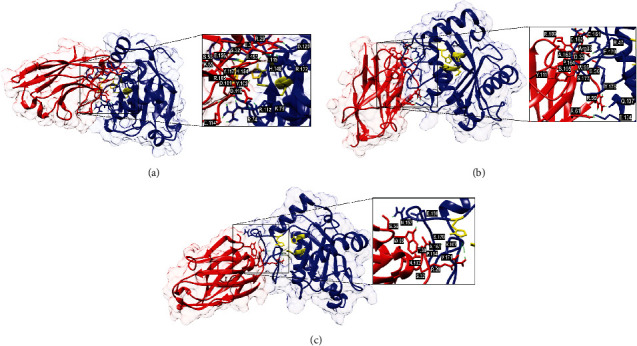
Molecular docking results showing the binding sites of VHHs (VHH47, VHH61, and VHH64) on the surface of BjussuMP-II. Cartoon representations of the BjussuMP-II VHH interaction structures (side view) covered by a translucent electronic surface, where the *α*-chains of BjussuMP-II are shown as blue ribbons and the VHH is in red. The active site of BjussuMP-II (His144, His148, His154, and Glu145) is represented in yellow. (a) Anti-BjussuMP-II VHH47 and BjussuMP-II. (b) Anti-BjussuMP-II VHH61 and BjussuMP-II. (c) Anti-BjussuMP-II VHH64 and BjussuMP-II. The interaction sites have been magnified to show the hydrogen bonds that formed between the amino acid residues.

The template chosen for the VHHs, with more than 70% similarity, was the camelid antibody fragment contained in the C-chain of the structure deposited in the PDB under code 4EJ1 [52]. Moreover, the selected template for BjussuMP-II showed 81% similarity to the crystallized structure of the metalloproteinase BaP1 from *Bothrops asper*, deposited in the PDB under code 1ND1 [17]. All modeled structures produced amino acids with Psi and Phi angles with more than 90% in highly favorable regions and no residues in forbidden regions. Also, the overlapping of the casts with the modeled targets resulted in an RMSD of less than 2 Å. In addition, all passed the Verify3D test, with 99% or more amino acids in regions above the cut-off value. After the dockings were performed, the most likely structural coordinates were extracted and analyzed (Figure 5 and Supplementary Figure 1).

As described by Marcussi et al. [20], the metalloproteinase domain of BjussuMP-II is found between histidines 144 and 154 (HELGHNLGMEH). Based on this, blind docking of anti-BjussuMP-II VHH47, anti-BjussuMP-II VHH61, and anti-BjussuMP-II VHH64 was performed against BjussuMP-II, resulting in scores of lower energy expenditure in the molecular dynamics of anti-BjussuMP-II VHH61 and the toxin (-809.5), followed by anti-BjussuMP-II VHH47 (-727.3) and anti-BjussuMP-II VHH64 (-596.0). The molecular docking of BjussuMP-II with anti-BjussuMP-II VHH47 presents interactions with His148 and His154, components of the binding region with the catalytic zinc cofactor, indicating that it interferes with blocking access to the substrate (Table 1).

## 4. Discussion

In order to overcome limitations of conventional serum therapy, better understanding of snake venom composition, and the identification of clinically important toxins, neutralizing epitopes shared among toxins and toxin synergism are the key to developing next-generation antivenoms [30, 54–56].

The synergistic effect between PLA_2_ and metalloprotease toxins in snakebites involving snakes of the *Bothrops* genus [11, 13, 54] supports the need for formulations of serum therapies with ample capacity to neutralize these major and medically important toxins in envenoming. Therefore, it is possible to infer that the selection of anti-metalloprotease VHHs, associated with VHHs previously selected against PLA_2_ (BthTX-I and BthTX-II) from *B. jararacussu* [27], might be an important tool in both the diagnosis and the treatment of snake envenoming by bothropic snakes. Thus, *Lama glama* VHHs that were active against the metalloprotease BjussuMP-II found in the venom of the snake *B. jararacussu* were chosen for this investigation.

In order to construct the VHH gene library, after purification and verification of the enzymatic activity of BjussuMP-II, the protein was used as an immunogen to induce an immune response in a *Lama glama* specimen. Unlike libraries from nonimmunized (naïve) donors, immune libraries from a donor immunized with the immunogen of interest have the advantage of refining the immune response, as well as improving the specificity and affinity of antibodies developed through immunization [53, 57, 58].

After undergoing weekly immunizations with sublethal doses and monitoring the antibody production titer with immunoenzymatic assays, on the 28^th^ day after immunization, a sample of the animal's blood was collected, with a maximum antibody detection titer in serum of 1 : 500,000 (*v* : *v*). This titer is satisfactory when compared to that described by Hmila et al. [59], who isolated a specific clone with an affinity of *K*_D_ = 55.8 nM for the neurotoxin AahI from venom of the scorpion *Androctonus australis* from the VHH library constructed by means of an animal response of 1 : 5000 (*v*/*v*) in an ELISA assay.

Phage display technology was used for the selection of camelid VHHs that were active against BjussuMP-II. After one round of biopanning, 52% of randomly selected clones recognized BjussuMP-II through an immunoenzymatic assay. This finding is considered promising since clones that particularly recognized the toxin and reduced its enzymatic activity were found after just one round of VHH selection against botulinum toxin, demonstrating affinity for the antigen similar to that of antibodies proposed as therapeutic agent for botulism [60]. Similarly, after one round of biopanning in the selection of anti-BthTX-I VHH from *Bothrops jararacussu* and anti-crotoxin VHH from *Crotalus durissus terrificus*, it was also able to get VHHs with high affinity with *K*_D_ = 53.8 nM [27] and *K*_D_ = 81.34 nM [28], respectively.

Among the 47 clones that recognized BjussuMP-II in ELISA, the clones that showed the best reactivity were selected and sequenced, identifying seven distinct profiles. In all the clones, the presence of amino acid substitutions in framework region 2 (FR2) (Y/F37) (E,Q44) (R45) (G/F/L47) was observed, a characteristic normally visualized in camelid VHHs, with the exception of anti-BjussuMP-II clone VHH53 which did not show the substitution at residue 44 but presented an amino acid that also has a hydrophilic character. This feature confers high solubility, thermal stability, and low propensity to form precipitates of VHH fragments [61, 62]. Unlike CDR3 in human VH (approximately 13 amino acids) [62], the CDR3 of the sequenced clones possessed between 16 and 19 amino acids. The longer CDR3 of VHHs can provide a larger available area for interaction with the surface antigen, reaching cavities inaccessible to conventional antibodies [62, 63]. Given the neglected profile of snakebites, new technologies need to be commercially viable for manufacturing and market distribution. Data indicate that innovative monoclonal antibody-based envenomation therapies can be manufactured at a cost comparable to or less than the current antiserum [64]. To this end, recombinant protein technology can be used to explore the scalable production capacity. Thus, the anti-Bjussu-MPII VHHs recombined in pET vectors, widely used for robust expression in *E. coli* capable of expressing the equivalent of up to 50% of heterologous recombinant protein of the total cellular proteins [65], can be produced in a scalable manner with low production costs.

Regarding the evaluation of the immunoreactivity of the different VHHs by ELISA, anti-BjussuMP-II VHH61 showed reactivity to *B. jararacussu* venom and cross-reactivity to a lesser extent against *B. brazili* venom, which may be related to the estimated concentration of 21.4% of PI class SVMPs in *B. brazili* [66]. Anti-BjussuMP-II VHH61 maintained its specificity for the *Bothrops* genus and did not recognize the *Crotalus* and *Lachesis* genera, a fact that can be explored in oligoclonal formulations as a strategy for the development of antivenoms [67, 68].

The proteolytic activity of BjussuMP-II on casein described above [20] was neutralized by anti-BjussuMP-II VHH61, indicating this VHH as an important tool with inhibitory potential. Determination of the cytotoxic effect of BjussuMP-II on endothelial cells demonstrated that this metalloproteinase induces damage to the integrity of tEND cells at a concentration of 20 *μ*g/200 *μ*L of BjussuMP-II. When cells were incubated with BjussuMP-II and anti-Bjussu MPII VHH61, at a 1 : 5 mass/mass ratio (toxin/VHH) for 48 h, there was a significant reduction, by 25% (*p* < 0.05), of LDH levels released in comparison to cells containing only BjussuMP-II. LDH is one of the most important enzymes in the cytoplasm, and under normal conditions, it cannot cross the cell membrane [41]. As a result, a rise in LDH is frequently reported as one of the hallmarks of cell membrane breakdown and necrosis [69]. In a study carried out by Franco et al. [41], just 5 *μ*g/mL of *B. jararaca* venom was able to cause the detachment of 32% of tEND cells within 2 h of incubation. This action on endothelial cells using a lower concentration of venom may be due to the amount of metalloprotease (42.8%) in the composition of this venom, in particular [70].

Anti-BjussuMP-II VHH61 along with other *β*-sheet-rich proteins can assume different conformational patterns, varying in parallel-antiparallel orientation, strand number and length, and level of twists [71]. These structural aspects account for morphological and spectral diversity in *β*-structures, which can be perceived on anti-BjussuMP-II VHH61 spectra in the form of a lower spectral amplitude at the 217 nm minimum; it is worth noting that this particular shape was previously seen in other VHHs [72] and proteins with *β*-sheet sandwich architecture.

The reliable CD analysis provided by the BeStSel algorithm [42] is evidenced by the fitted curve reaching a NRMSD of 0.01, which considers the orientation and twists of *β*-sheets in protein aggregates and amyloid fibrils. Therefore, the analysis generated a robust secondary structure estimation for anti-BjussuMP-II VHH61, with a distribution of elements in complete agreement with the theoretical model for anti-BjussuMP-II VHH61, corroborating the molecular modelling results.

Taking into account the range of fixed wavelengths (200-205 nm) used in the thermal titration of VHHs in previous studies [73–76], anti-BjussuMP-II VHH61's thermal stability was monitored at 202 nm. In the reverse temperature ramp, anti-BjussuMP-II VHH61 did not refold, possibly due to an aggressive aggregation propensity and the formation of amyloidogenic segments upon unfolding, thus preventing recovery to its original conformation.

Although the molecular coupling of the toxin with anti-BjussuMP-II VHH61 and anti-BjussuMP-II VHH64 by modeling and molecular docking does not show the expected interactions between the zinc-binding groups, it is possible to visualize important links in the loop between h4 and h5, which, according to the computational model, has two disulfide bridges (Cys159-Cys183 and Cys161-Cys166), which aid in structural stability, a determinant for the activities performed by this protein [20]. Anti-BjussuMP-II VHH61 in particular showed specific binding of an amino acid from its CDR3 with Cys166, a component of this disulfide bridge. In this loop region, the presence of His154 can also be observed, represented as a zinc-binding motif, and together with the other histidines in the metalloproteinase domain, it is essential for catalytic activity [77]. The loop between the two *α*-helices also has the CIMP sequence, where a methionine residue (met-turn) is found, which is another structure in charge of maintaining the stability of the molecule, favoring the binding of His154 to zinc, as well as comprising a hydrophobic base for the active site of the groups [15, 17, 78]. Anti-BjussuMP-II VHH61 also exhibited bonds with the loop between *β*-sheets 4 and 5, which has a disulfide bridge (Cys117-Cys199) with the *α*-helix loop h5, being responsible for stabilizing the C-terminal region of the protein [32]. In addition to this interaction, this VHH is bound to the *α*-helix h4, demonstrating a promising binding potential for possible neutralization of part of the enzymatic activity, since His144, His148, and Glu145, amino acids responsible for binding to catalytic zinc, are found in this helix [20, 79]. The aforementioned points of interaction may be associated with the ability of anti-BjussuMP-II VHH61 to reduce LDH release by the endothelial cell incubated with the metalloprotease.

## 5. Conclusion

Investigation of snakebite pathophysiology and snake venom components has allowed for the identification and characterization of clinically relevant toxins, as well as conserved epitopes and toxin synergism. This knowledge, together with antibody engineering techniques, has guided the development of tools for next-generation antivenoms. Single-domain antibodies are small compounds that have the potential to be utilized in antivenom formulations. Furthermore, VHHs are a promising tool for neutralizing local damage induced by snakebite envenoming, strengthening the goal that qualifies them as a serum immunotherapy adjuvant. Given the important role played by the metalloprotease BjussuMP-II in the toxicity of *B. jararacussu* snakebites, anti-BjussuMP-II VHHs become essential tools for the development of next-generation bothropic antivenoms. Further research is needed in this area to confirm the likelihood of action against metalloproteases from different classes, due to the presence of a conserved prodomain and a catalytic domain in this group.

There is an urgent need for antivenom therapies that improve the effectiveness of local treatment, leading to a trend in antivenom research aimed at reducing the use of animal immunizations, replacing them with recombinant monoclonal antibody production technologies and omics technologies such as venomics, antivenomics, and toxicovenomics. The data presented, though preliminary, point to a potential for therapeutics employing camelid single-domain antibodies.

## Figures and Tables

**Table 1:** Molecular interactions between amino acid residues of VHHs and BjussuMP-II.

VHH	VHH domain	Amino acids		BjussuMP-II domain	Distance (Å)
		VHH	BjussuMP-II		
Anti-BjussuMP-II VHH47	CDR2	Ser-56	Glu-156	Loop between h4 and h5	1.9
	CDR3	Arg-105	His-154	Zinc catalytic site	1.9/2.0/2.5
	CDR3	Arg-105	Glu-153	Loop between h4 and h5	1.9/2.1
	CDR2	Lys-55	Glu-153	Loop between h4 and h5	1.7
	CDR3	Thr-103	His-148	Zinc catalytic site	2.3
	CDR1	Arg-29	Asp-120	Loop between *β*4 and *β*5	1.8/1.9
	CDR1	Ser-32	Ile-115	Loop between *β*4 and *β*5	1.8
	FR1	Glu-3	Arg-122	Loop between *β*4 and *β*5	1.8
	CDR3	Thr-103	Arg-112	Loop between *β*4 and *β*5	1.8
	CDR3	Asp-101	Arg-112	Loop between *β*4 and *β*5	2.4
	CDR3	Asp-101	Arg-74	*α*-Helix h3	2.0/1.7
	CDR3	Asp-114	Arg-74	*α*-Helix h3	1.8/1.8
	CDR3	Asp-116	Lyz-78	*α*-Helix h3	1.8/1.8
Anti-BjussuMP-II VHH61	CDR3	Arg-109	Cys-161	Loop between h4 and h5	1.7
	CDR3	Arg-109	Ala-163	Loop between h4 and h5	1.7
	CDR3	Tyr-111	Pro-164	Loop between h4 and h5	1.9
	CDR1	Arg-33	Asp-162	Loop between h4 and h5	1.8/1.8
	CDR1	Arg-33	Glu-179	Loop between h4 and h5	1.9/1.9
	CDR3	Ser-105	Ser-177	Loop between h4 and h5	1.8
	CDR2	Trp-55	Ser-177	Loop between h4 and h5	2.3
	CDR1	Asp-32	Arg-47	*α*-Helix h2	2.0/1.9
	CDR2	Ser-56	Arg-47	*α*-Helix h2	1.7
	FR3	Arg-61	Glu-134	Loop between *β*5 and h4	2.0/1.2/1.8
	CDR2	Arg-59	Glu-134	Loop between *β*5 and h4	1.9/1.7
	CDR2	Arg-59	Gln-137	*α*-Helix h4	1.7
	CDR2	Arg-59	Tyr-178	Loop between *β*4 and *β*5	2.0
	CDR2	Ser-56	Arg-47	*α*-Helix h2	1.7
Anti-BjussuMP-II VHH64	CDR1	Arg-29	Val-171	Loop between h4 and h5	1.8
	CDR1	Arg-29	Val-167	Loop between h4 and h5	2.1/2.3
	CDR1	Thr-30	Ser-170	Loop between h4 and h5	2.3
	CDR1	Ser-32	Pro-164	Loop between h4 and h5	1.8
	CDR2	Trp-55	Glu-156	Loop between h4 and h5	2.0
	CDR3	Asn-102	Ala-163	Loop between h4 and h5	2.0
	CDR2	Ser-56	Asn-157	Loop between h4 and h5	1.9

## Data Availability

The data of this manuscript will be made available by the corresponding author on a reasonable request.

## Supplementary Materials

Supplementary Materials Modeling and molecular docking of the anti-BjussuMP-II VHHs. In addition to VHH modeling and molecular docking of the anti-*Bjussu*MP-II VHHs (VHH47, VHH61, and VHH64), binding site interactions of VHH34, VHH53, VHH78, and VHH79 on the surface of BjussuMP-II were also analyzed. The interaction sites have been magnified to show the hydrogen bonds that formed between the amino acid residues (Supplementary Figure 1). Supplementary Figure 1: molecular docking results showing the binding sites of VHHs on the surface of BjussuMP-II. Cartoon representations of the BjussuMP-II VHH interaction structures (side view) where the *α*-chains of BjussuMP-II are shown as blue ribbons and the VHH is in red. A: Anti-BjussuMP-II VHH34 and BjussuMP-II. B: Anti-BjussuMP-II VHH53 and BjussuMP-II. C: Anti-BjussuMP-II VHH78 and BjussuMP-II. D: Anti-BjussuMP-II VHH79 and BjussuMP-II. The interaction sites have been magnified to show the hydrogen bonds that formed between the amino acid residues.
