# Supplementary material for: Anti-Metalloprotease P-I Single-Domain Antibodies: Tools for Next-Generation Snakebite Antivenoms
Source: Biomed Res Int. 2022 Jul 19;2022:2748962. doi: 10.1155/2022/2748962 (PMC9325618; doi:10.1155/2022/2748962)
Supplement: Supplementary Materials — Modeling and molecular docking of the anti-BjussuMP-II VHHs. In addition to VHH modeling and molecular docking of the anti-BjussuMP-II VHHs (VHH47, VHH61, and VHH64), binding site interactions of VHH34, VHH53, VHH78, and VHH79 on the surface of BjussuMP-II were also analyzed. The interaction sites have been magnified to show the hydrogen bonds that formed between the amino acid residues (Supplementary Figure 1). Supplementary Figure 1: molecular docking results showing the binding sites of VHHs on the surface of BjussuMP-II. Cartoon representations of the BjussuMP-II VHH interaction structures (side view) where the α-chains of BjussuMP-II are shown as blue ribbons and the VHH is in red. A: Anti-BjussuMP-II VHH34 and BjussuMP-II. B: Anti-BjussuMP-II VHH53 and BjussuMP-II. C: Anti-BjussuMP-II VHH78 and BjussuMP-II. D: Anti-BjussuMP-II VHH79 and BjussuMP-II. The interaction sites have been magnified to show the hydrogen bonds that formed between the amino acid residues. [file 2748962.f1.docx]

**
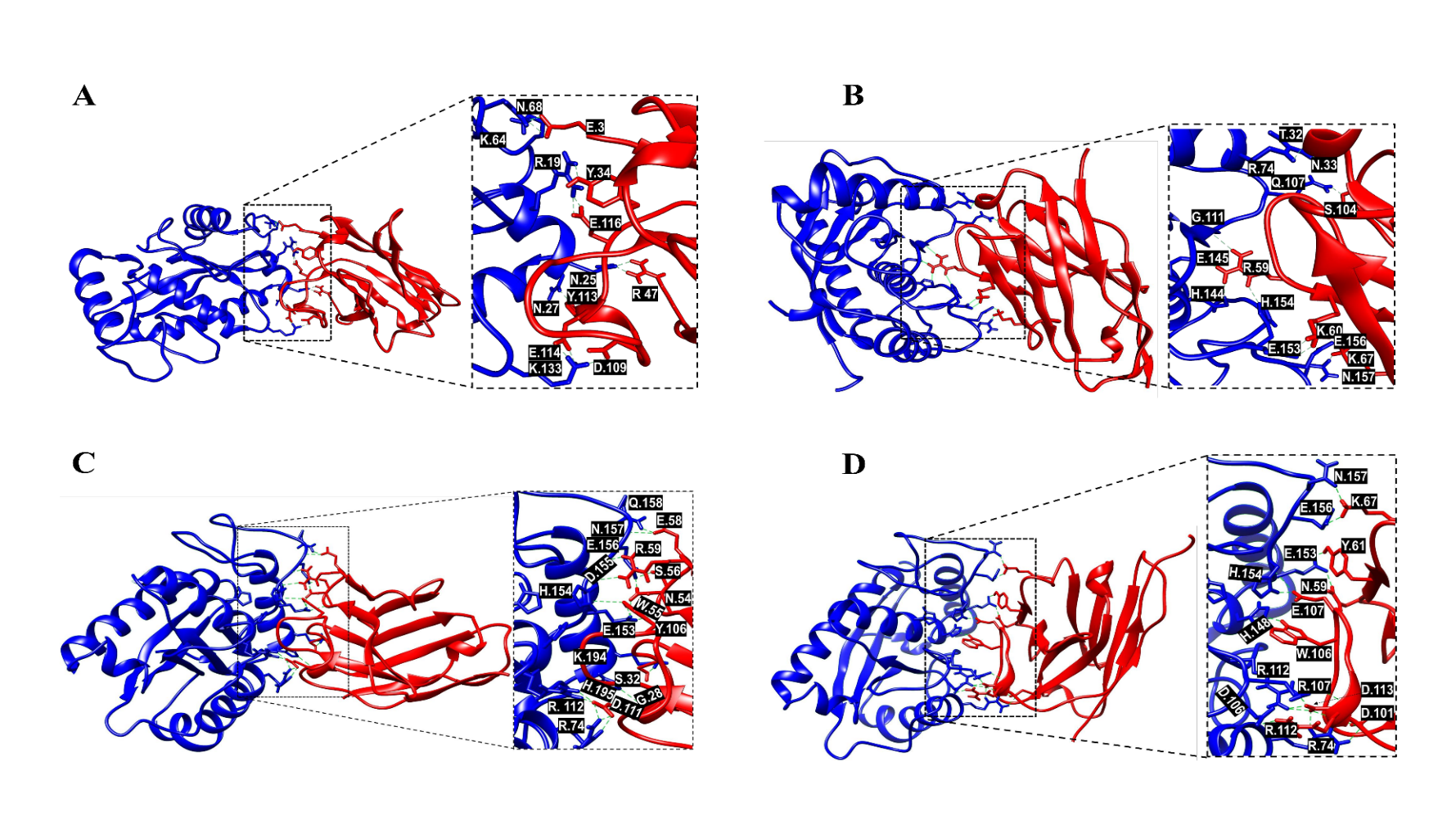
Supplementary Material**

**Supplementary Figure 1.** Molecular docking results showing the binding sites of VHHs on the surface of BjussuMP-II. Cartoon representations of the BjussuMP-II VHH interaction structures (side view) where the α-chains of BjussuMP-II are shown as blue ribbons and the VHH is in red. A: Anti-BjussuMP-II VHH34 and BjussuMP-II. B: Anti-BjussuMP-II VHH53 and BjussuMP-II. C. Anti-BjussuMP-II VHH78 and BjussuMP-II. D: Anti-BjussuMP-II VHH79 and BjussuMP-II. The interaction sites have been magnified to show the hydrogen bonds that formed between the amino acid residues.
